# An influence of dew point temperature on the occurrence of *Mycobacterium tuberculosis* disease in Chennai, India

**DOI:** 10.1038/s41598-022-10111-4

**Published:** 2022-04-12

**Authors:** Rajendran Krishnan, Kannan Thiruvengadam, Lavanya Jayabal, Sriram Selvaraju, Basilea Watson, Muniyandi Malaisamy, Karikalan Nagarajan, Srikanth P. Tripathy, Ponnuraja Chinnaiyan, Padmapriyadarsini Chandrasekaran

**Affiliations:** 1 ICMR-National Institute for Research in Tuberculosis, Chennai, India; 2 DTO, NTEP, Greater Chennai Corporation, Chennai, India

**Keywords:** Climate sciences, Diseases, Medical research, Mathematics and computing

## Abstract

Climate factors such as dew point temperature, relative humidity and atmospheric temperature may be crucial for the spread of tuberculosis. This study was conducted for the first time to investigate the relationship of climatic factors with TB occurrence in an Indian setting. Daily tuberculosis notification data during 2008–2015 were generated from the National Treatment Elimination Program, and analogous daily climatic data were obtained from the Regional Meteorological Centre at Chennai city, Tamil Nadu, India. The decomposition method was adopted to split the series into deterministic and non-deterministic components, such as seasonal, non-seasonal, trend and cyclical, and non-deterministic climate factors. A generalized linear model was used to assess the relation independently. TB disease progression from latent stage infection to active was supported by higher dew point temperature and moderate temperature. It had a significant association with TB progression in the summer and monsoon seasons. The relative humidity may be favored in the winter and post-monsoon. The water tiny dew droplets may support the TB bacterium to recuperate in the environment.

## Introduction

Tuberculosis (TB) is a leading and tenth world’s deadliest infectious killer caused by *Mycobacterium tuberculosis*. Almost 4000 people lose their lives, and nearly 28,000 people fall ill every day due to this preventable and curable disease^1^. TB is an airborne infectious disease and is transmitted commonly through cough droplet nuclei of minute size (1–5 microns in diameter) expelled by persons with PTB disease. These tiny particles can stay up in air for several hours, and persons may become infected when they inhale droplet nuclei containing tubercle bacilli^2^.

Studies concerning seasonal variations in TB have employed predication models to show the seasonal and trend effects on the occurrence of TB^3^. Time-series analysis of the monthly incidence of smear-positive pulmonary tuberculosis in China during 2004–2015 showed that cases topped in the period between January and March^4^. Studies conducted have also shown seasonal variation in TB occurrence with peaks in spring and summer and low prevalence in winter, underscoring the links to low immunity when weakening immunity that may induce the TB reactivation risk in these seasons because possible insight was that vitamin D deficiency in winter^5^. In Japan, the age disparity of TB infection had a seasonal impact, and people aged > 15 years peaked in summer. The monthly case flow varied by age and sex, and it was observed that cases peaked from June to December for females and declined for male patients between September and December. The trend revealed that seasonal-specific risk factors pertaining to TB are needed to better understand it^6^. Childhood TB cases were correlated with the presence of more rainfall^7^. TB progression was found to be influenced by low temperature, humidity and rainfall, showing a linear relation with relative humidity^8^.

Several ecological environmental climate factors, such as temperature (Temp), dew point temperature (DPT) and relative humidity (RH), may be crucial in enabling the sustenance and spread of Mycobacteria tuberculosis and determining the temporal seasonal changes in the occurrence of disease over years. Dew points are water in the form of tiny droplet nuclei that appear on thin, exposed objects in the morning or evening due to condensation. As the exposed surface cools by radiating its heat, atmospheric moisture condenses at a rate greater than that at which it can evaporate, resulting in the formation of water droplets^9^. However, existing studies supporting this view are relatively scant.

In this background, the aim of the present study is to investigate the relational impact climatic factors on TB occurrence by utilizing a novel analytical method and valuable data obtained from National Tuberculosis Elimination Program (NTEP) Chennai, India. This is the first report from India that addresses the relationship between climate factors and the progression of TB infection from the latent stage to active in humans over the sequence of time.

## Result

A total of 5777 TB cases occurred in different parts of Chennai during the period of 551 days (between October 2007–2015) as per the NTEP, Chennai. The results showed that the outcome variable of DPT was the highest evening DPT (> − 2 °C to − 4 °C day variation), and 819 TB cases occurred in 77 days at a rate of 11 cases/day. The moderate (> 0 °C to − 2 °C day variation) evening DPT, 1820 TB cases were befallen in 195 days at the rate of 9.3cases/day. On day zero, 270 cases occurred in 23 days at a rate of 12 cases/day. The highest morning (> 2–4 °C Day variation) DPT was in 57 days, of which 679 TB cases occurred at a rate of 12 cases/day. The moderate (> 0 °C to 2 °C day variation) morning DPT occurred on 199 days, of which 2189 TB cases occurred at a rate of 11 cases/day. In winter, the evening high DPT (> 0 °C to − 2 °C) and morning high DPT (0 °C to 6 °C) were predominant, with an average DPT, RH and Temp of 20–22 °C, 70–75%, and 26–27 °C, respectively.

The highest TB patient occurrence was moderately high during the morning. In summer, the evening high DPT (> 0 °C to − 6 °C) and morning high DPT (0 °C to 2 °C) were predominant when the average DPT, RH and Temp were 24.8–25.3 °C, 64–73%, and 30–33 °C, respectively.

The highest patient flow was observed during the evening and morning day variations of DPT > 0 °C to − 2 °C and > 0 °C to 2 °C, respectively. Moreover, the mild variation of 0.5 °C throughout the day). During the monsoon season, the evening high DPT (> 0 °C to − 6 °C) and morning high DPT (0 °C to 2 °C) were predominant, with average DPT, RH and Temp values of 24.3–25 °C, 70–78% and 30–32 °C, respectively. The highest patient flow was observed during the evening and morning day variation of DPT > − 6.0 °C to 2 °C. The DPT showed a mild variation of 0.7 °C throughout the day.

**Figure 1:**
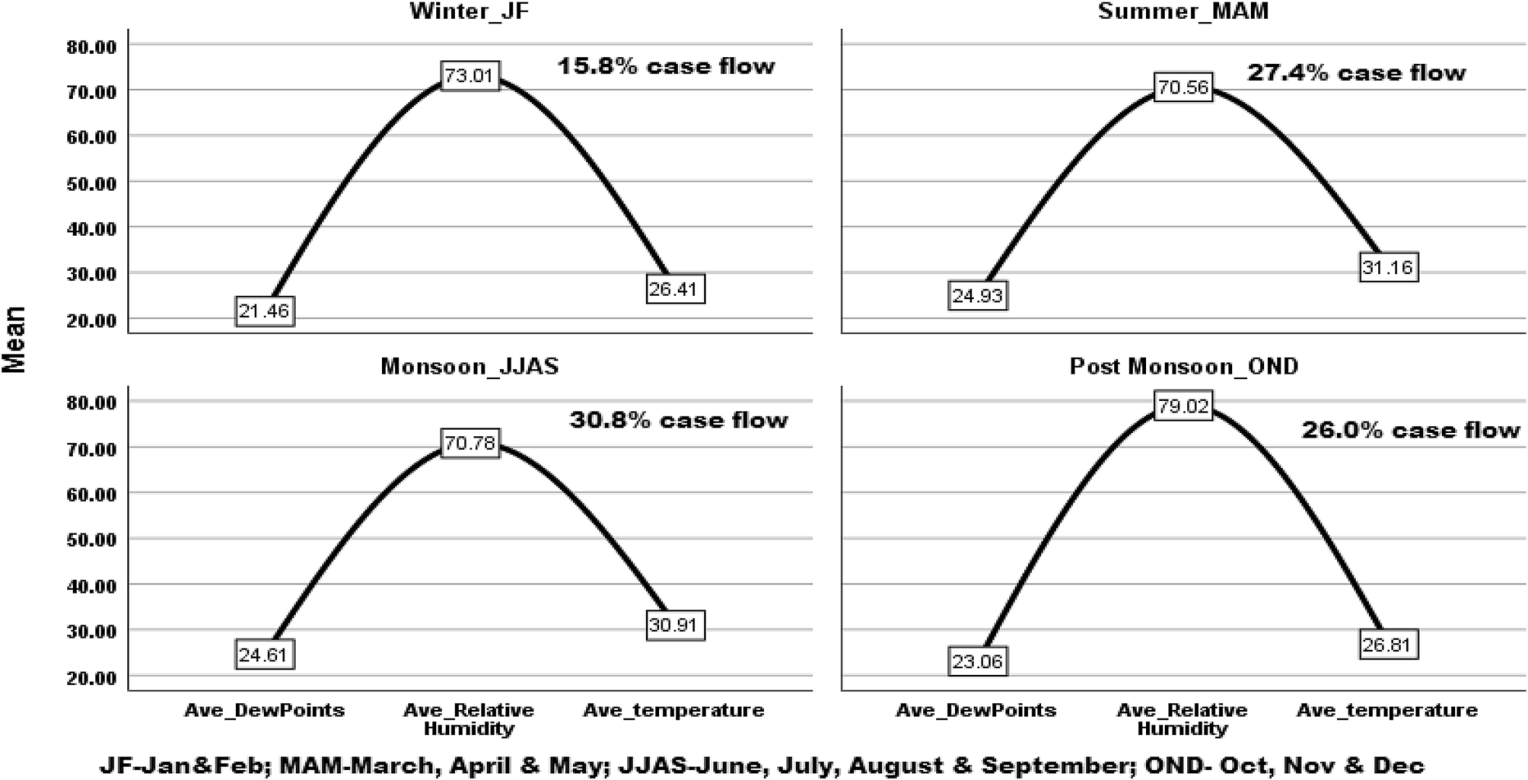
High patient flow in the monsoon and summer seasons and a high drop in the winter season. *Method*: Group line chart focusing climate factors. *Results*: The patient flow in all seasons and variability in climate factors were explained.

During the post-monsoon season, the evening high DPT (> 0 °C to − 2 °C) and morning high DPT (0 °C to 6 °C) were predominant, with average DPT, RH and Temp of 21–24 °C, 71–88%, and 26–28 °C, respectively. The highest patient flow was observed during the evening and morning day variation of DPT > 0 °C to 2 °C. An almost fifty percent drop in patient flow was observed in the winter season (Table 1, Fig. 1).

**Table 1:** Seasonal variation and TB notification respective days Dew point temperature.

Seasons	Day’s variation of Dew point temperature				
**Winter TB cases (n = 915)**					
	< − 2 °C to ≤ − 4 °C n = 5	< 0 °C to − 2 °C n = 154	NV n = 60	> 0 °C–2 °C n = 513	> 2 °C–high n = 183
RH	62.00 ± 0.00	74.50 ± 4.45	68.50 ± 4.39	74.21 ± 6.07	70.19 ± 5.80
DPT	19.05 ± 0.00	22.28 ± 1.49	20.62 ± 1.26	21.96 ± 1.38	19.69 ± 1.53
Temp	26.40 ± 0.00	26.82 ± 1.11	26.24 ± 0.50	26.65 ± 0.95	25.46 ± 0.92
**Summer TB cases (n = 1583)**					
	n = 226	n = 647	n = 89	n = 555	n = 66
RH	64.20 ± 5.01	72.09 ± 6.45	73.16 ± 3.65	72.60 ± 4.30	56.54 ± 11.67
DPT	24.82 ± 1.54	25.28 ± 1.41	25.21 ± 1.24	24.84 ± 1.31	22.24 ± 1.40
Temp	33.37 ± 1.28	31.27 ± 1.68	31.12 ± 1.77	29.94 ± 1.74	32.77 ± 3.95
**Monsoon TB cases (n = 1777)**					
	n = 550	n = 618	n = 79	n = 464	n = 69
RH	69.51 ± 9.13	72.57 ± 9.94	78.29 ± 6.55	70.57 ± 9.22	57.51 ± 8.30
DPT	24.73 ± 1.24	24.97 ± 1.43	24.98 ± 1.00	24.26 ± 1.64	22.26 ± 1.67
Temp	31.81 ± 1.80	31.01 ± 1.64	29.62 ± 1.66	29.87 ± 1.38	31.23 ± 0.71
**Post-monsoon TB cases (n = 1502)**					
	n = 41	n = 401	n = 42	n = 657	n = 361
RH	71.61 ± 6.91	81.24 ± 8.54	88.19 ± 6.91	81.72 ± 6.26	71.41 ± 8.90
DPT	22.02 ± 1.24	23.60 ± 1.73	24.34 ± 1.04	23.95 ± 1.66	20.79 ± 2.47
Temp	27.87 ± 2.54	26.64 ± 2.70	27.95 ± 0.52	27.16 ± 1.38	26.10 ± 1.51

**Figure 2:**
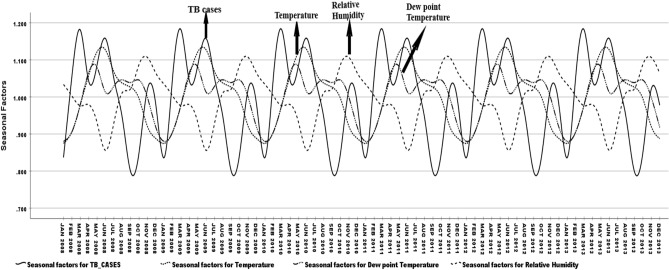
Climate factors influence TB infection in the seasons. *Method*: Individual line chart focusing on climate factors influencing TB infection. *Results*: Seasonal climate factors influence TB infection.

Yearly, three seasonal peaks were found to be associated with peaks of TB occurrence. The first peak was observed in March and April, while the dew point temperature was almost constant throughout the day when the DPT ranged from 24.3 to 25 °C DPT and 30 to 33 °C temperature with a 2 °C day variation when the RH was 70% to 78% with a 2%-day variation between the morning and evening. The second TB peak was observed in May and June with a high temperature (31.5 °C), moderate RH (71%) and highest dew point temperature (25 °C). Similarly, during November and December, TB progression was observed with moderate temperature (27 °C) and highest RH (80%) with moderate dew point temperature (23 °C) (Fig. 2).

**Figure 3:**
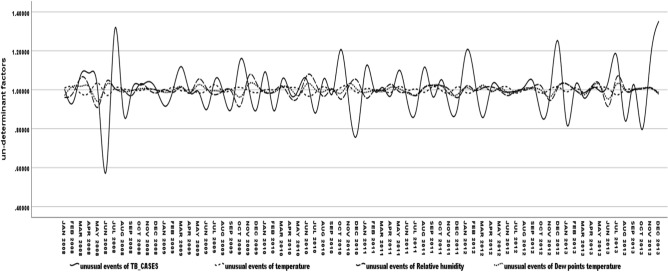
Dew point temperature and temperature influence unusual TB infection in seasons. *Method*: Individual line chart focusing on climate factors influencing unusual TB infection. *Results*: Identifying unusual TB infections on seasonal climate factor influences.

For both summer and monsoon season. The progression of TB infection occurred above 24 °C DPT with < 70% RH and temperature between 32 and 33 °C when evening DPT is 4 °C higher than days morning, Decomposition procedure, seasonal adjustment factors revealed seasonality of TB diseases progressive relation correlated with temperature (correlation ρ = 0.404; p < 0.001) and dew point temperature (correlation ρ = 0.618; p < 0.001). Figure 3 shows that the unusual case flows in the seasons are influenced by the dew point temperature and air temperature.

### Seasonal factors

Seasonal factors model SF-TB = SF-DPT + SF-Temp + SF-RH with intercept (β = 6.14 (4.77–7.50): p < 0.001)) showed TB progression in the season had significant relation to high dew point Temperature. SF-DPT (β = 4.32(4.77–7.50): p < 0.001)) in the summer (β = 0.15(0.09–0.22): p < 0.001)) and had association with monsoon (β = 0.04(− 0.02 to 0.10): p = 0.240)) while the temperature (SF-Temp) and relative humidity (SF-RH) were moderately low, because DPT and Temperature had inverse relation in nature (Eq. 1). In summer, RH and Temp were not influenced as much compared to DPT.

### Seasonal adjusted factors

The non-seasonal model SAF-TB = SAF-DPT + SAF-Temp + SAF-RH with intercept (β = 177.74 (12.43–343.04): p < 0.033)) showed that TB progression in the non-seasonal had a significant relation to dew point temperature. In addition, SAF-DPT (β = 9.35 (2.40–16.31): p < 0.001)) in the seasons (β = 67.65 (62.80–72.50): p < 0.001)). Non-seasonal factors of DPT, Temp, and RH contributed significantly to the progression of TB positivity among patients in the seasons (Eq. 2).

### Trend cycle

Trend model T&C-TB = T&C-DPT + T&C-Temp + T&C-RH with intercept (β = 341.16 (217.97–464.36): p < 0.001)) showed that TB progression was in increasing trend both season and non-seasons while increasing the dew point temperatures T&C-DPT (β = 15.55(9.58–21.53): p < 0.001)) and decreased significantly the temperatures T&C-Temp (β = − 13.87 (− 18.47 to − 9.28): p < 0.001)) and relative humidity T&C-RH (β = − 3.22(− 4.59 to − 1.86): p < 0.001)) in the season (Eq. 3).

### Error (non-deterministic factor (NDF))

NDF-TB = NDF-DPT + NDF-Temp + NDF-RH with intercept (β = 0.79(− 2.64 to 4.22): p = 0.647)) showed that TB progression had positive association in the non-deterministic events of NDF-NPT (β = 1.35(− 1.57 to 4.28): p = 0.358)), dew point temperature as well as NDF-Temp (β = 0.02(− 3.25 to 0.43): p = 0.271) temperature in the seasons(β = 1.02(0.96–1.07): p < 0.001) significantly (Eq. 4). Overall non-deterministic case flow of TB patients was influenced by the dew point temperature and air temperature in the summer and monsoon seasons and relative humidity predominantly in the winter and post-monsoon seasons.

## Discussion

Tuberculosis is an air borne disease. *Mycobacterium tuberculosis* survival in the environment is stable, and tubercle bacilli are transported in fine droplets from infected persons’ lungs into the air by cough. The bacilli can survive up to 6 months outside the environment without direct sunlight. They also settle so often in dusty and dark areas^10,11^. M tuberculosis, surviving at temperatures lower than 100 °C, did not constantly kill M tuberculosis; furthermore, it projected the survival of 50% and 25% of the organisms after heat inactivation at 95 °C in a dry heat block with durations of 20 and 30 min, respectively^12^. It evidenced that MTB could survive even up to 95 °C. Active and smear-positive PTB cases were reported in Chengdu, China, in the seasonality pattern, of which the influence of climatological factors was evidenced during high peaks in March and April^13^. The peak season changed over the winter between 2006 and 2016 to the summer between 2006 and 2012 and further shifted to spring between 2013 and 2016. Notified tuberculosis in Korea also showed seasonality and confirmed that climate would be the influential factor irrespective of seasons throughout years and period in between because it explored the different seasonal peaks of TB infection at different time intervals. Thus, it is necessary to evaluate climate factors related to the seasonality of TB to better control and eliminate this global epidemic^14^.

The present study revealed that the dew point temperature is positively related to TB disease progression in different seasons. Dew points are pure water in the form of tiny droplets that appear on thin, exposed objects in the morning or evening due to condensation in the atmosphere. People are infected by inhaling *Mycobacterium tuberculosis* in droplet form, which is transmitted from one person to another through tiny air droplets. Both summer and monsoon season Dew Point temperatures were high irrespective of season. In realistic scenarios, as the above studies showed that *Mycobacterium tuberculosis* is so stable to survive in the environment, dew point droplets may support bacilli to survive actively in seasons. This could be because in the dusty environment, *Mycobacterium tuberculosis* may become active by tiny dew point droplets with no direct sunlight.

The outcome of this study showed that the dew point temperature was highly significantly related to TB progressive infection in the seasons. The tiny dew point micro droplets may help the MTB revive and carry the infection to humans in the season when they are immunosuppressed by seasonal weather^15^. The DPT, Temp and RH are interrelated, and the dew points move an air temperature in the atmosphere, which becomes the moisture content temperature. Both air temperature and dew point temperature have inverse relationships with each other if they are equal to 100% RH, which is the highest atmospheric pressure that stimulates condensation.

A review study revealed that TB reporting rates increase with temperature and decline with altitude^16^. The peak TB case notification rate corresponded with the end of the dry season in the two agrarian regions of Ethiopia. Regional variations in TB seasonality thus might require attention to geographic-specific TB case-finding strategies because the role of climate factors in bacterial survival is a very important component^17^. The study also suggested that TB prevention and control interventions, such as efforts to increase community TB awareness and contact tracing, should consider seasonal variation.

These findings were emphasized to highlight why the relation of dew point temperature to TB infection is important. The dew is water in the form of tiny droplets that appear thin due to condensation. It is always lower than (or equal to) the air temperature. *Mycobacterium tuberculosis* is in an environment so stable to survive even in a dusty environment, and it may be activated by tiny dew point droplets. Plant- and human-pathogenic bacteria can be preserved in pure water or phosphate-buffered saline (PBS) for several years. Gram-plus bacteria appear to survive better in PBS than in water^18^. Generally, in the local summer, the climate is so uncomfortable that it seems to have higher relative humidity, but it is dew point temperature because when the air temperature is high, relative humidity never be high rather dew point temperature is so progresses to bring down the air temperature. The bacteria survived at high air temperatures (> 35 °C) and were unable to survive; instead, they survived at higher DPTs. At the dew point, atmospheric moisture condensation results in the formation of water droplets, where these bacteria may propagate and comfortably survive because the comfort level is > 17 °C at a maximum of approximately 25 °C. Bacteria may grow across a wide range of temperatures, from very cold to very hot. All human pathogens are mesophilic and grow best at moderate temperatures, neither too hot nor too cold^19,20^. A systematic review including 49 studies revealed that the seasonality of the disease’s progression of tuberculosis infection had consistent peaks. It occurred in spring or summer and reached fall in winter, which was proven through multiple studies. Stronger seasonality was associated with younger patients, extrapulmonary disease, and latitudes farther from the equator, where El Niño and La Niña originate every 1- and half-years or 2-year interval^21^.

Tuberculosis notification from 2005 to 2014 in Xinjiang, China, showed apparent seasonal variation with a peak in March and a trough in October. This specific study expressed that its peak in winter was due to overcrowding indoors^22^. The spatiotemporal pattern of TB incidence presented high geographical variations, with two main hot spots with a peak in late winter meteorological measures^23^.

### Adapted analytical procedure

Generally, in statistics, handling in the error terms and unusual peaks in the health research to be emphasized to include in the analysis and predict. In the parametric models, the unusual events are named outliers, which will be omitted automatically in the model. In this study, the decomposition methodology was adapted to handle both systematic and non-deterministic events. The decomposition procedure was adapted for climate relation analysis to determine the independence of the influence of climate factors on TB infection, which is the theory of time series analysis. The idea of decomposing a time series into deterministic and non-deterministic components. It is predominantly used for time series analysis tools to impart forecasting models that provide a structured method for a time series forecasting problem to alleviate modeling complexity and specifically to best explore each of these components and to identify specific patterns of series in the period. Even the organism transmits infection based on evidenced climate factor variability irrespective of seasons and years. This was the exclusive sphere of the climate factors and health reliability, which could be handled by our opted methodology. Handling long seasonality data, the innovative methodology was developed to elevate misinterpretation in the routine existing methodology.

The study guided by the original characteristics of the data-driven goal accomplishment split a time series into seasonal, trend and random residual time series. All the results showed a relation with TB infection, such as seasonal components designated seasonal influences that evidenced significant peak seasonality and long-term progression of the series, which are all deterministic components in the series. There is evidence of only non-changing seasonality throughout the period, where the dew point temperature in the summer and beginning of monsoon seasons has a strong influence, while the dew point temperature has the highest influence. The randomness of time series can detect non-deterministic components in the series, i.e., unusual events in the series, such as sudden peaks or downward trends, where environmental and climate factors may only influence the high morbidity of TB. In Holy Kerbala, Iraq. The seasonality of PTB incidence peaked in spring and winter over the period 2016 to 2018 and showed marginal decreasing trends after winter^24,25^.

It has been found that climate factors influence TB diagnosis deviance in different seasons, even the performance of QuantiFERON-TB Gold In-Tube (QFT-GIT) assays in a temperate climate for the diagnosis of TB infection, which was higher in summer and spring and lower in autumn and winter^26^. Evidence of seasonal variation changes the response to antigen stimulation in the test of interferon gamma release assays (IGRAs) for TB diagnosis^27^. Climate changes, a potentially modifiable factor often related to nutritional and vitamin D (VD) deficiencies due to a lack of sun exposure, led to VD deficiency in household contact (HHC) with the lowest solar radiation exposure during winter/spring. Spring enrollment, compared with other seasons, was the principal risk factor for latent TB infection in HHC^28,29^. It has been shown that sufficient vitamin D intake enhances the immune response against *Mycobacterium tuberculosis*.

Clinically heterogeneous conditions with similar seasonal phenotypes were present for TB patients in season in a hospital-based study in New York State from 2008 to 2011^30^. The outbreaks of infectious disease in seasonality revealed that climate and environment have a significant risk of pathogens of interest, and the effects can be complementary. Major environmental factors that can affect this are solar radiation, primarily acting through ultraviolet radiation (UVR), and its subsequent control of vitamin D production^31^. Climatic variables included average temperature and average ultraviolet radiation. Vitamin D deficiency among humans was strongly associated with the lowest solar radiation exposure in the winter/spring seasons in cold countries; VD is a highly influential factor supporting the immune system of humans.

The roles of temperature, dew point temperature and relative humidity are the prime factors in changing the seasons. The bacterium is also adapting its survival in that climatic environment; if the unusual climate rises up and down, the bacterium may mutate in another form, which may impact drug resistance. Many of the studies have shown evidence of seasonal changes that affect active and smear-positive PTB, not only that the performance of diagnostic tools for TB assays varies in the seasons. Addressing all climate influence has customized human survival, leading to high dependency on these factors; not every change in the human body is avertable from the influence of climate factors either directly or indirectly because all living beings are vulnerable to climate change. The seasons cause immune suppression in their body system, such as high peaks of winters and summer. Children are also so vulnerable to catch the cold in winter and wet seasons because of the high moisture contents due to dew points on the environment, which has been imposed by the temperature up and down variation. In the summer, moderate temperatures with high dew points may hit the immune suppressive mode of older people, while the bacterium finds the pathway to activate its virulence to become an active TB.

## Conclusion

Climate factors are highly influential on TB disease progression in seasons and are interrelated in both sea and land climate-changing activities in the atmospheric environment. This research very strongly has comprehended that TB disease progression from latent stage infection to active seems to be supported by higher dew point temperature and moderate temperature in summer and monsoon seasons, and relative humidity may be favoured in the winter and post-monsoon seasons. The water tiny dew droplets may support MTB to revive and execute the environment.

## Methods

In this study, daily individual TB *case* data were collected manually from patient records maintained at NTEP centers, Chennai, for 2008 to 2015. Analogous daily climatic data, which included local RH, temperature and DPT, were obtained from the Regional Meteorological Centre at Chennai. The data for the years 2008–2013 were considered for this analysis to determine the patterns of TB infection among the treated patients. The data were quality checked for missing information and mined for central tendency and dispersion with box plots for distributional identity. Data characteristics of each variable clarity were assessed and plotted in a sequential curve. Month wise data were restructured for tuberculosis-positive cases along with the mean monthly RH, temperature and DPT, of which the decomposed factors were derived using a time series model and analyzed individually using a generalized linear model (GLM) that included linear regression to predict TB progression over seasons influenced by climate factors^32^.

State TB Officer of National Treatment Elimination Program, Chennai city had granted permission to collect these data for research purpose only through then commissioner of greater Chennai Corporation. It was secondary data collection for this project; all methods were carried out in accordance with relevant guidelines and regulation i.e. institutional scientific advisory and Ethics committees were approved to takeaway further.

### Innovative method for climate factors

For climatic factors, the day relative humidity, temperature, and dew point temperature [*i.e*., morning (max)–evening (min)] were created as outcome variables for each factor as per the Regional Meteorological Centre, Chennai data that monitored weather in Tamil Nadu. The following climatic factors were recoded.

The dew point temperature was recoded as follows

- 1 =  ≤ − 2.01 °C to − 4 °C = evening high
- 2 =  < 0 °C to − 2 °C = evening moderately high
- 3 = constant,
- 4 =  > 0 °C to 2 °C = morning moderately high
- 5 =  > 2 °C to 5 °C = morning high.

The Air temperature was recoded as follows

- 1 =  ≤ 5 °C = mild variation,
- 2 =  > 5 °C to 10 °C = moderate variation
- and 3 =  > 10 °C to 15 °C = high variation.

The Relative humidity was recoded as follows

- 1 =  < 0%–evening high,
- 2 = constant,
- 3 =  > 0% to 10% mild variation,
- 4 =  > 10% to 20% moderate variation
- 5 =  > 20% to 30% high variation.

The seasons were coded as 1 = Winter: January and February, 2 = Summer: March, April and May, 3 = Monsoon: June, July, August and September and 4 = Post-monsoon: October, November, and December. The decomposition procedure was relevant to identify the actual causative factors instead of mean factors^33^. For this procedure, monthly TB cases were used for analysis. The sequential curve showed an upward trend highlighting the distinct seasonal pattern of TB cases in Chennai city. The existing seasonal pattern permitted a series trend that suggested predominant factors were additive seasonality, i.e., the occurrence of changes in season with similar patterns over the period.

### Seasonal decomposition procedure

The theory of time series analysis shows decomposing a time series into deterministic and non-deterministic components. Time series decomposition splits a time series into seasonal, trend and random residual time series. The trend and the random time series can both be used to detect irregularities of the events during the period.

**Figure 4:**
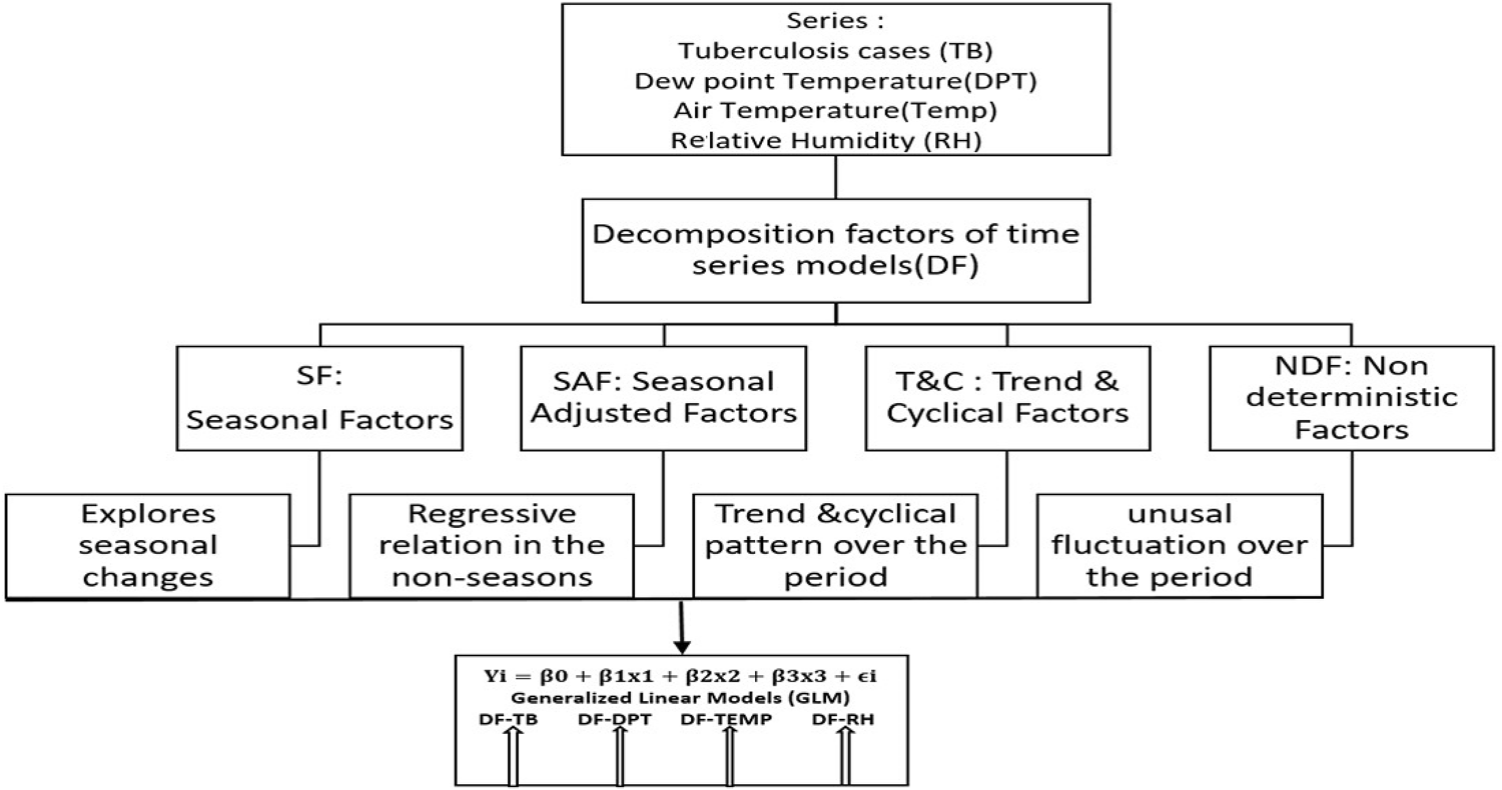
Schematic analysis flow of GLM for the decomposition factor of the time series model. *Method*: organigram of analytical methodology. *Results*: The decomposition procedure and GLM analysis are explained.

The timeline occurrence of TB patients, Temp, DPT and RH were considered for time series analysis decomposed methodology that contained a series as combination of level, trend, seasonality and undeterministic (noise) components. The procedure determined the individual influence in the series and explored the potential influence of climate factors. Two major components in the time series were systematic components, which consisted of three subcomponents: (1) seasonal adjusted factors (SAF-(level)), (2) trend and cycle (T&C-(trend)), (3) seasonal factors (SF-(seasonality)), and (4) non-systematic components, which consisted of non-deterministic factors (NDF-(noise)) (Fig. 4). The model adopted in this study presented trends and seasonality with a set of predictor variables for RH, temperature, and dew point temperature. A stationary time series had a mean and variance that were essentially constants over time.

Ensuring the variability of relative humidity and dew points in relation to tuberculosis infection.

The seasonal factors explored only seasonal patient flow associations. However, the seasonally adjusted factors showed an unseasonal patient flow relation. Trend and cyclical factors identified the pattern of increasing and decreasing patient flow in the seasonal and non-seasonal. Undeterministic (noise) factors revealed the unusual patient flow relation to climate factors. Each factor of all four was fitted in a generalized linear model with TB patients as the dependent variable and all other climate factors as exploratory variables with seasonality factorization. The model effects were considered foremost to intercept in the model with hybrid Fisher scoring to obtain the maximum likelihood estimation. The model effect analysis was a type III method with a 95 percent confidence interval, likelihood ratio, chi-square statistics and profile likelihood confidence interval to determine the model information and to elevate the favorable factors such as RH, temperature and DPT ranges through seasons by GLM. This procedure was more feasible to estimate the relation between TB and climate factors in the seasons than the average values.

Generalized linear models (GLMs) are a broad class of models that include linear regression (Table 2) as follows:

**Table 2:** Generalized linear model exploring the TB and climate factor relation.

Climate factors	Parameter (SE)	95% CI	Wald X^2^	P-values
**Seasonal factors in the series**				
Intercept	6.139 (0.69)	4.77–7.50	79.867	< 0.001*
SF-DPT	4.32 (0.76)	2.80–5.84	31.86	< 0.001*
SF-Temp	− 4.96 (0.83)	− 6.62 to − 3.31	35.57	< 0.001*
SF-RH	− 4.50 (0.58)	− 5.66 to − 3.34	59.25	< 0.001*
**Relation to seasons**				
Intercept	0.950 (0.02)	0.90–0.99	1619.02	< 0.001*
Winter	− 0.004 (0.04)	− 0.08 to 0.07	0.011	0.91
Summer	0.153 (0.03)	0.09–0.22	21.10	< 0.001*
Monsoon	0.037 (0.03)	− 0.02 to 0.10	1.30	0.24
Post-monsoon	Reference factor			
**Seasonal adjusted factors in series (SAF)**				
Intercept	177.74 (83.23)	12.43–343	4.56	0.033*
SAF-DPT	9.35 (3.50)	2.40–16.31	7.13	0.008*
SAF-Temp	− 6.29 (2.87)	− 11.10 to − 0.59	4.80	0.028*
SAF-RH	− 2.01 (0.81)	− 3.61 to − 0.40	6.17	0.013*
**Relation to seasons**				
Intercept	67.65 (2.44)	62.80–72.50	768.27	< 0.001*
Winter	3.90 (3.85)	− 3.76 to − 11.57	1.02	0.312
Summer	2.07 (3.45)	− 4.78 to − 8.93	0.36	0.550
Monsoon	2.06 (3.22)	− 8.47 to − 4.35	0.41	0.520
Post-monsoon	Reference factor			
**Trend and cyclical events in the series (T&C)**				
Intercept	341.16 (62.03)	217.97–464.36	30.25	< 0.001*
T&C-DPT	15.55 (3.01)	9.58 to 21.53	26.71	< 0.001*
T&C-Temp	− 13.87 (2.31)	− 18.47 to − 9.28	35.96	< 0.001*
T&C-RH	− 3.22 (0.64)	− 4.59 to − 4.59	21.47	< 0.001*
**Relation to seasons**				
Intercept	66.74 (1.53)	63.71 to 69.77	1910.07	< 0.001*
Winter	4.30 (2.41)	− 0.50 to − 9.09	3.17	0.075
Summer	2.16 (2.16)	− 2.13 to − 6.45	1.00	0.317
Monsoon	− 0.24 (2.02)	− 4.25 to − 3.77	0.01	0.905
Post-monsoon	Reference factor			
**Undeterminant factor (error) in the series (UDF)**				
Intercept	0.79 (1.72)	− 2.64 to − 4.22	0.21	0.647
UDF-DPT	1.35 (1147)	− 1.57 to − 4.28	0.84	0.358
UDF-Temp	0.02 (1.55)	− 3.07 to − 3.10	0.900	0.992
UDF-RH	− 1.16 (1.05)	− 3.25 to − 0.43	1.21	0.271
**Relation to seasons**				
Intercept	1.02 (0.03)	0.96–1.07	1335.10	< 0.001*
Winter	− 0.01(0.04)	− 0.10 to − 0.07	0.08	0.772
Summer	0.01 (0.14)	− 0.08 to − 0.07	0.02	0.888
Monsoon	− 0.03 (0.04)	− 0.11 to − 0.04	0.82	0.365
Post-monsoon	Reference factor			

*DPT* Dew point temperature, *Temp* temperature, *RH* relative humidity.

*Statistically significant.

$$Yi=\beta 0+\beta 1x1+\beta 2x2+\beta 3x3+\epsilon i$$

### Seasonal factors

$$SF\to TB=6.14+4.32*SF\to DPT-4.96*SF\to Temp-4.50*SF\to RH+0.006$$

Season:

1 $$ \begin{aligned} & Seasons=0.95-0.00*Winter+0.15*Summer+0.04*Monsoon+0.010 \\ & \quad \quad \quad {\text{Post-monsoon is set to zero by default in the model}}  \end{aligned}$$

### Seasonal adjusted factors

$$SAF\to TB=177.73+9.35*SAF\to DPT-6.29*SAF\to Temp-2.01*SAF\to RH+101.37$$

Season:

2 $$\begin{aligned} & Seasons=67.65+3.90*Winter+2.07*Summer-2.06*Monsoon+107.23 \\ & \quad \quad \quad {\text{Post-monsoon is set to zero by default in the model}}  \end{aligned}$$

### Trend-cycle

$$T\&C\to TB=341.16+15.55 \, * \,T\&C\to DPT-13.87 \,* \,T\&C\to Temp-3.22 \,*\,T\&C\to RH+28.23$$

Season:

3 $$\begin{aligned} & Seasons=66.74+4.29*Winter+2.16*Summer-0.24*Monsoon+41.98 \\ & \quad \quad \quad  {\text{Post-monsoon is set to zero by default in the model}}\end{aligned}$$

### Error (non-deterministic factor (NDF))

$$NDF\to TB=0.79+1.35*UDF\to DPT+0.02*UDF\to Temp-1.16*UDF\to RH+0.014$$

Season:

4 $$\begin{aligned} &Seasons=1.02-0.01*Winter-0.01*Summer-0.03*Monsoon+0.01  \\ & \quad \quad \quad  {\text{Post-monsoon is set to zero by default in the model}}\end{aligned}$$

## References

[1] https://www.who.int/campaigns/world-tb-day/world-tb-day-2021#:~:text=TB%20remains%20one%20of%20the,lives%20since%20the%20year%202000.

[2] https://www.cdc.gov/tb/education/corecurr/pdf/chapter2.pdf.

[3] Liao Z, Zhang X, Zhang Y, Peng D (2019). Seasonality and trend forecasting of tuberculosis incidence in Chongqing, China. Interdiscip. Sci..

[4] Mao Q, Zeng C, Zheng D, Yang Y (2019). Analysis on spatial-temporal distribution characteristics of smear positive pulmonary tuberculosis in China, 2004–2015. Int. J. Infect. Dis..

[5] Sumi A, Kobayashi N (2019). Time-series analysis of geographically specific monthly number of newly registered cases of active tuberculosis in Japan. PLoS ONE.

[6] Manabe T, Takasaki J, Kudo K (2019). Seasonality of newly notified pulmonary tuberculosis in Japan, 2007–2015. BMC Infect. Dis..

[7] Jaganath D, Wobudeya E, Sekadde MP, Nsangi B, Haq H, Cattamanchi A (2019). Seasonality of childhood tuberculosis cases in Kampala, Uganda, 2010–2015. PLoS ONE.

[8] Kuddus MA, McBryde ES, Adegboye OA (2019). Delay effect and burden of weather- related tuberculosis cases in Rajshahi province, Bangladesh, 2007–2012. Sci. Rep..

[9] Rajendran K, Sumi A, Bhattachariya MK, Manna B, Sur D, Kobayashi N, Ramamurthy T (2011). Influence of relative humidity in *Vibrio cholerae* infection: A time series model. Indian J. Med. Res..

[10] Tuberculosis in the Workplace Marilyn J. Field, Editor, Committee on Regulating Occupational Exposure to Tuberculosis, Division of Health Promotion and Disease Prevention, 26–30.

[11] http://www.tbonline.info/posts/2016/3/31/how-tb-spread-1/.

[12] Zwadyk P, Down JA, Myers N, Dey MS (1994). Rendering of mycobacteria safe for molecular diagnostic studies and development of a lysis method for strand displacement amplification and PCR. J. Clin. Microbiol..

[13] Chengdu, China, the active and smear-positive PTB case reported in the seasonality pattern, peaking in March and April, with apparent links to social dynamics and climatological factors.

[14] Kim EH, Bae JM (2018). Seasonality of tuberculosis in the Republic of Korea, 2006–2016. Epidemiol. Health..

[15] John W (1917). Trask, climate and tuberculosis: The relation of climate to recovery. Public Health Rep. (1896–1970).

[16] Gelaw YA, Yu W, Magalhães RJS, Assefa Y, Williams G (2019). Effect of temperature and altitude difference on tuberculosis notification: A systematic review. J. Glob. Infect. Dis..

[17] Gashu Z, Jerene D, Datiko DG, Hiruy N, Negash S, Melkieneh K, Bekele D, Nigussie G, Suarez PG, Hadgu A (2018). Seasonal patterns of tuberculosis case notification in the tropics of Africa: A six-year trend analysis in Ethiopia. PLoS ONE.

[18] Liao CH, Shollenberger LM (2003). Survivability and long-term preservation of bacteria in water and in phosphate-buffered saline. Lett. Appl. Microbiol..

[19] https://courses.lumenlearning.com/boundless-microbiology/chapter/temperature-and-microbial-growth/.

[20] Jabir RA, Rukmana A, Saleh I, Kurniawati T (2018). The existence of *Mycobacterium tuberculosis* in microenvironment of bone. Mycobacterium Res. Dev..

[21] Tedijanto C, Hermans S, Cobelens F, Wood R, Andrews JR (2018). Drivers of seasonal variation in tuberculosis incidence: Insights from a systematic review and mathematical model. Epidemiology.

[22] Wubuli A, Li Y, Xue F, Yao X, Upur H, Wushouer Q (2017). Seasonality of active tuberculosis notification from 2005 to 2014 in Xinjiang, China. PLoS ONE.

[23] Guo C, Du Y, Shen SQ, Lao XQ, Qian J, Ou CQ (2017). Spatiotemporal analysis of tuberculosis incidence and its associated factors in mainland China. Epidemiol. Infect..

[24] Mohammed SH, Ahmed MM, Al-Mousawi AM, Azeez A (2018). Seasonal behavior and forecasting trends of tuberculosis incidence in Holy Kerbala, Iraq. Int. J. Mycobacteriol..

[25] Mao Q, Zhang K, Yan W, Cheng C (2018). Forecasting the incidence of tuberculosis in China using the seasonal autoregressive integrated moving average (SARIMA) model. J. Infect. Public Health..

[26] Tebruegge M, Curtis N, Clifford V, Fernandez-Turienzo C, Klein N, Fidler K, Mansour S, Elkington P, Morris-Jones S (2018). Seasonal variation in the performance of QuantiFERON-TB Gold In-Tube assays used for the diagnosis of tuberculosis infection. Tuberculosis.

[27] Griffin ML, Agarwal S, Murphy MZ, Teeter LD, Graviss EA (2018). Influence of seasonality and circulating cytokines on serial QuantiFERON discordances. Tuberc. Res. Treat..

[28] Balcells ME, García P, Tiznado C, Villarroel L, Scioscia N, Carvajal C, Zegna-Ratá F, Hernández M, Meza P, González LF, Peña C, Naves R (2017). Association of vitamin D deficiency, season of the year, and latent tuberculosis infection among household contacts. PLoS ONE.

[29] Coussens AK (2017). The role of UV radiation and vitamin D in the seasonality and outcomes of infectious disease. Photochem. Photobiol. Sci..

[30] Haimovich JS, Venkatesh AK, Shojaee A, Coppi A, Warner F, Li SX, Krumholz HM (2017). Discovery of temporal and disease association patterns in condition-specific hospital utilization rates. PLoS ONE.

[31] You S, Tong YW, Neoh KG, Dai Y, Wang CH (2016). On the association between outdoor PM(2.5) concentration and the seasonality of tuberculosis for Beijing and Hong Kong. Environ. Pollut..

[32] Senja Passimaa. *Humidity Expressed as Dewpoint Temperature*. Reprinted with permission of Vaisala, Inc. originally published in the 154/2000 issue of the Vaisala News.

[33] Hardin JW, Hilbe JM (2007). Generalized Linear Models an Extension.

